# Supplementary material for: Origin and evolution of GATA2a and GATA2b in teleosts: insights from tongue sole, Cynoglossus semilaevis
Source: PeerJ. 2016 Mar 21;4:e1790. doi: 10.7717/peerj.1790 (PMC4806627; doi:10.7717/peerj.1790)
Supplement: Figure S1 [file peerj-04-1790-s001.pdf]

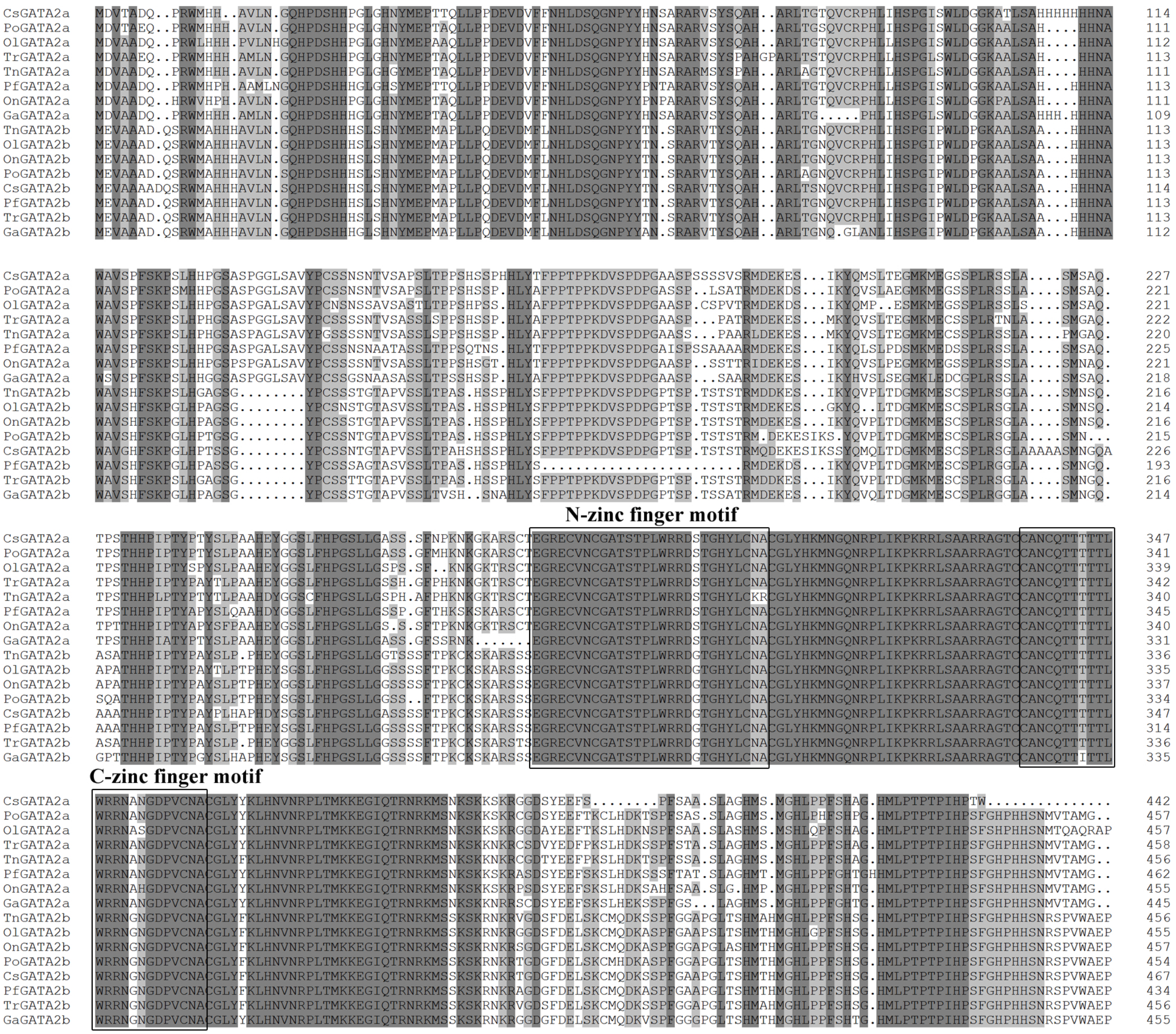
N-zinc finger motif
C-zinc finger motif
