# Supplementary figures and images for: Origin and evolution of GATA2a and GATA2b in teleosts: insights from tongue sole, Cynoglossus semilaevis
Source: PeerJ. 2016 Mar 21;4:e1790. doi: 10.7717/peerj.1790 (PMC4806627; doi:10.7717/peerj.1790)

A

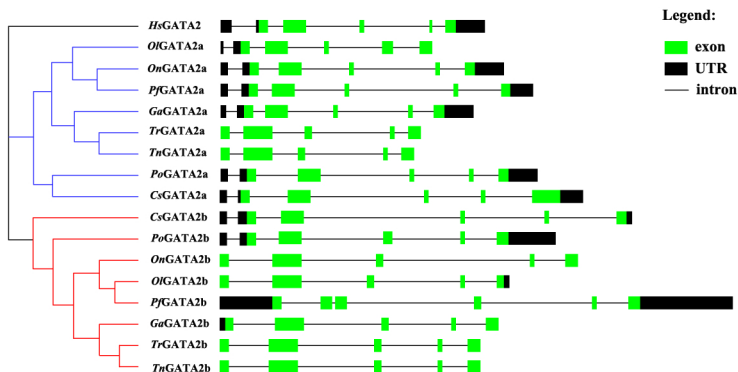

B

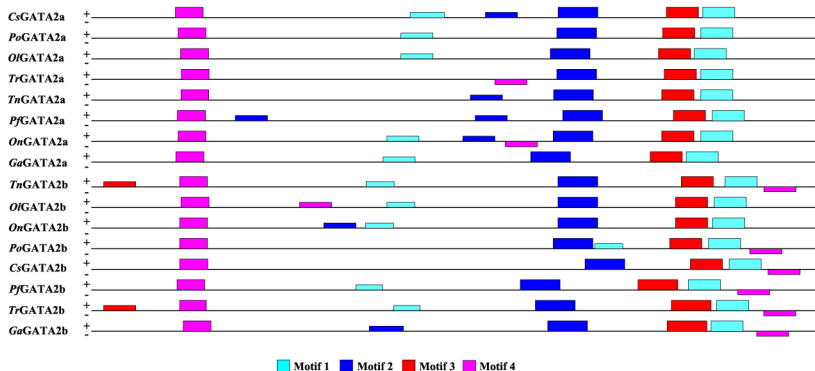

Supplement: Figure s2 — (A) ML phylogenetic tree and exon–intron structures of the GATA2 genes. Box: exon; lines: introns. The lengths of boxes and lines are scaled based on gene length. (B) MEME motif search results. Conserved motifs are indicated in numbered color boxes. [file peerj-04-1790-s002.pdf]

**A**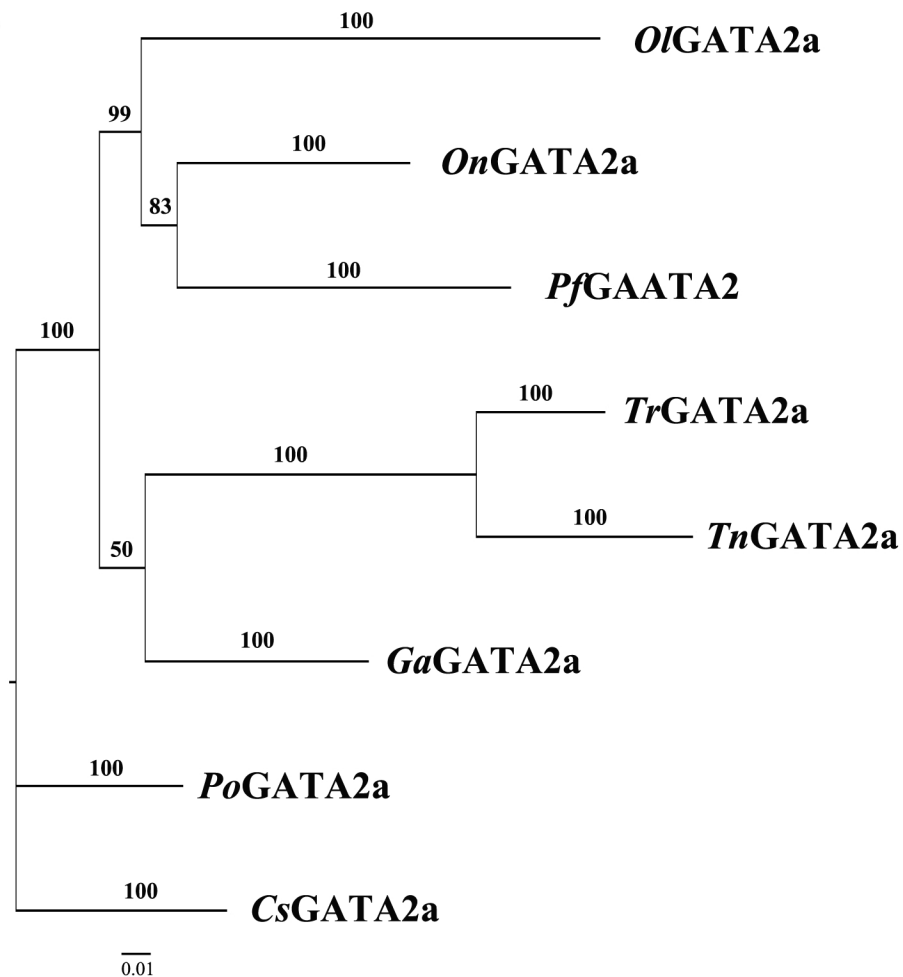**B**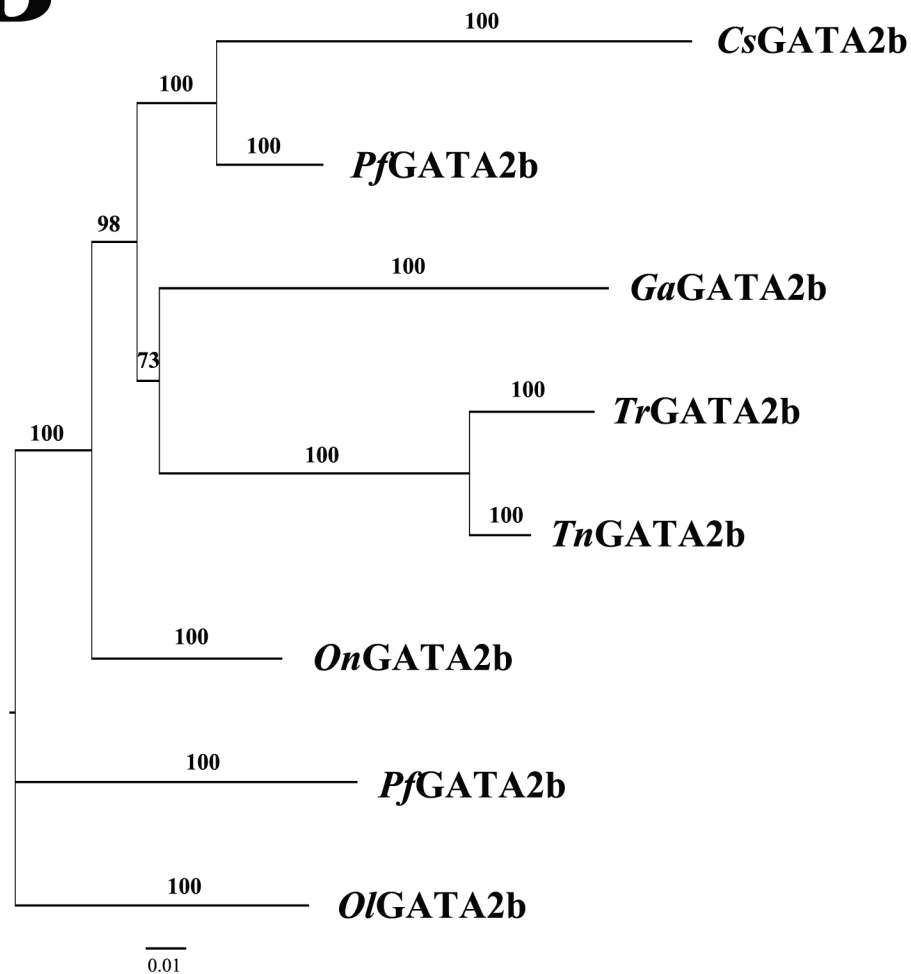

Supplement: Figure S3 — (A) Phylogenetic tree constructed based on GATA2a sequences by using MrBayes with the TPM2uf+G model to assess selection pressure; MCMC = 200,000. (B) Phylogeny for site model constructed based on GATA2b sequences by using MrBayes with the TIM2+I model (MCMC = 200,000). [file peerj-04-1790-s003.pdf]

**A**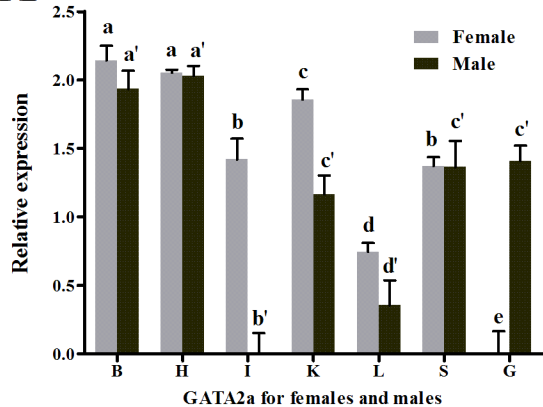**B**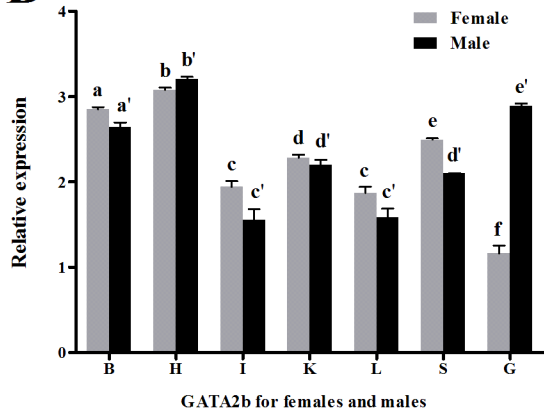

Supplement: Figure S4 [file peerj-04-1790-s004.pdf]
