# Supplementary material for: Origin and evolution of GATA2a and GATA2b in teleosts: insights from tongue sole, Cynoglossus semilaevis
Source: PeerJ. 2016 Mar 21;4:e1790. doi: 10.7717/peerj.1790 (PMC4806627; doi:10.7717/peerj.1790)
Supplement: Table S1 [file peerj-04-1790-s005.docx]

**Table S1** Database ID of the sequences used in this study.

| Species | Gene | Accession number |
| --- | --- | --- |
| *Poecilia formosa*  *Takifugu rubripes*  *Oryzias latipes*  *Gasterosteus aculeatus*  *Tetraodon nigroviridis*  *Oreochromis niloticus*  *Nematostella vectensis*  *Homo sapiens*  *Mus musculus*  *Gallus gallus* | GATA2a  GATA2b  GATA2a  GATA2b  GATA2a  GATA2b  GATA2a  GATA2b  GATA1  GATA2a  GATA2b  GATA3  GATA4  GATA5  GATA6  GATA1  GATA2a  GATA2b  GATA3  GATA4  GATA5  GATA6  GATA  GATA1  GATA2  GATA3  GATA4  GATA5  GATA6  GATA1  GATA2  GATA3  GATA4  GATA5  GATA6  GATA1  GATA2  GATA3  GATA4  GATA5  GATA6 | Ensembl: ENSPFOT00000003754  Ensembl: ENSPFOT00000003221  Ensembl: ENSTRUT00000035095  Ensembl: ENSTRUT00000025931  Ensembl: ENSORLT00000001689  Ensembl: ENSORLT00000009689  Ensembl: ENSGACT00000012700  Ensembl: ENSGACT00000001029  Ensembl: ENSTNIT00000010303  Ensembl: ENSTNIT00000017851  Ensembl: ENSTNIT00000014247  Ensembl: ENSTNIT00000006468  Ensembl: ENSTNIT00000019696  Ensembl: ENSDARG00000017821  Ensembl: ENSTNIT00000018102  Ensembl: ENSONIT00000025596  Ensembl: ENSONIT00000023989  Ensembl: ENSONIT00000025651  Ensembl: ENSONIT00000022612  Ensembl: ENSONIT00000017057  Ensembl: ENSONIT00000000582  Ensembl: ENSONIT00000011829  GenBank: AY465174.1  GenBank: AAH09797.1  GenBank: AAH51272.1  GenBank: XP_005252500.1  GenBank: AAI43480.1  GenBank: NP_536721.1  GenBank: NP_005248.2  GenBank: NP_032115.1  GenBank: NP_032116.4  GenBank: P23772.1  GenBank: NP_032118.2  GenBank: NP_032119.2  GenBank: NP_034388.2  GenBank: NP_990795.1  GenBank: NP_001003797.1  GenBank: NP_001008444.1  GenBank: NP_001280035.1  GenBank: NP_990752.1  GenBank: NP_990751.1 |
