# Supplementary material for: Origin and evolution of GATA2a and GATA2b in teleosts: insights from tongue sole, Cynoglossus semilaevis
Source: PeerJ. 2016 Mar 21;4:e1790. doi: 10.7717/peerj.1790 (PMC4806627; doi:10.7717/peerj.1790)
Supplement: Table S2 [file peerj-04-1790-s006.docx]

**Table S2** Primers used for qRT-PCR.

| Primer name | Sequence |
| --- | --- |
| Cs-GATA2a-RT-Fw  Cs-GATA2a-RT-Rv  Cs-GATA2b-RT-Fw  Cs-GATA2b-RT-Rv | GTGCTCAACGGACAACA  GTGAGTCCAGGTGGTTAAAG  CAACGAGGACATCGGTTATC  GTACGAACGCTTCCAAATAGA |
